# Striking a Balance between Work and Play: The Effects of Work–Life Interference and Burnout on Faculty Turnover Intentions and Career Satisfaction

**DOI:** 10.3390/ijerph19020809

**Published:** 2022-01-12

**Authors:** Sheila A. Boamah, Hanadi Y. Hamadi, Farinaz Havaei, Hailey Smith, Fern Webb

**Affiliations:** 1 Faculty of Health Sciences, School of Nursing, McMaster University, 1280 Main Street West, Hamilton, ON L8S 4K1, Canada; 2 Department of Health Administration, Brooks College of Health (Building 39), University of North Florida, Jacksonville, FL 32224, USA; h.hamadi@unf.edu (H.Y.H.); n01379505@unf.edu (H.S.); 3 School of Nursing, University of British Columbia, Vancouver, BC V6T 2B5, Canada; farinaz.havaei@ubc.ca; 4 Department of Surgery-Jacksonville, Center for Health Equity & Engagement Research, Urban Health Alliance of Jacksonville, College of Medicine, University of Florida, Jacksonville, FL 32209, USA; fern.webb@jax.ufl.edu

**Keywords:** burnout, career satisfaction, COVID-19, faculty shortage, nursing faculty, turnover intentions, work environment, work–life interference

## Abstract

Background: The interactions between work and personal life are important for ensuring well-being, especially during COVID-19 where the lines between work and home are blurred. Work–life interference/imbalance can result in work-related burnout, which has been shown to have negative effects on faculty members’ physical and psychological health. Although our understanding of burnout has advanced considerably in recent years, little is known about the effects of burnout on nursing faculty turnover intentions and career satisfaction. Objective: To test a hypothesized model examining the effects of work–life interference on nursing faculty burnout (emotional exhaustion and cynicism), turnover intentions and, ultimately, career satisfaction. Design: A predictive cross-sectional design was used. Settings: An online national survey of nursing faculty members was administered throughout Canada in summer 2021. Participants: Nursing faculty who held full-time or part-time positions in Canadian academic settings were invited via email to participate in the study. Methods: Data were collected from an anonymous survey housed on Qualtrics. Descriptive statistics and reliability estimates were computed. The hypothesized model was tested using structural equation modeling. Results: Data suggest that work–life interference significantly increases burnout which contributes to both higher turnover intentions and lower career satisfaction. Turnover intentions, in turn, decrease career satisfaction. Conclusions: The findings add to the growing body of literature linking burnout to turnover and dissatisfaction, highlighting key antecedents and/or drivers of burnout among nurse academics. These results provide suggestions for suitable areas for the development of interventions and policies within the organizational structure to reduce the risk of burnout during and post-COVID-19 and improve faculty retention.

## 1. Introduction

Burnout is rampant among the academic workforce and nursing faculty are no exception. The high prevalence of burnout among faculty is attributed to the characteristics of the work environment including excessive workloads, time demands, high expectations and fierce competition, and lack of support and poor leadership [1,2]. The ongoing rise of COVID-19-pandemic-related stressors and unrelenting/accelerating work expectations has further exacerbated burnout among faculty [3]. Emerging studies suggest that the pandemic has blurred the boundaries between individuals’ work and personal life, leading to more unequal work–life balances amongst faculty members [4,5]. From a psychological perspective, the COVID-19-induced shift to virtual/online formats for teaching and learning creates additional stress and challenge to faculty members due to the increased demands for student support and workload in the absence of face-to-face interactions [5]. Moreover, the demands of the pandemic and its innumerable effects have placed additional pressure on faculty and, in many ways, limiting their ability to initiate research without decreasing the pressure and expectation to publish [6], thus further increasing risks of burnout.

The high prevalence of burnout is grounds for concern, as it has deleterious effects on career longevity and job turnover, which further worsens the ongoing nursing workforce shortages and poses a serious risk for mental health challenges, faculty well-being, and quality of life [7]. It is critical that burnout and turnover of nursing faculty is addressed as it could bottleneck the number of nurses that would be able to receive training, restricting the number of clinical nurses in the future. While factors that lead to burnout have been widely discussed in the organizational and healthcare literature among clinical nurses [8,9,10], few studies have investigated the antecedents or predictors of burnout among nursing faculty [4]. To our knowledge, no study has comprehensively examined academic nurses’ work–life balance and association with burnout and turnover intentions during the pandemic. Given that the main causes of burnout are deeply rooted within the work environment, it is important to understand how organizational factors predisposes faculty to burnout especially in the context of COVID-19 to effectively mitigate the risks.

To extend evidence from past research, the present study investigated the mediating effects of burnout on the relationships between work–life interference and turnover intentions and career satisfaction among nursing faculty. Understanding these relationships is crucial to inform evidence-based interventions, strategies, and policies to address the dimensions of burnout in academic settings.

**Figure 1:**
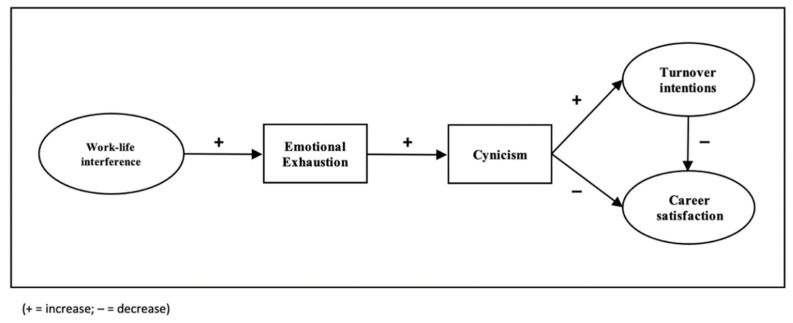
Hypothesized study model proposing the mediating effect of burnout (emotional exhaustion and cynicism) on the relationship between work-life interference, turnover intensions, and career satisfaction.

In this study, we integrated Greenhaus et al.’s [11] theory of work–life interference and Leiter and Maslach’s [12] burnout model as the theoretical framework to examine personal and workplace factors that influence nursing faculty retention outcomes (see Figure 1). 

### 1.1. Work–Life Interference

Work–life interference (or lack of balance) is defined as an inter-role conflict where work demands make it such that one is unable to concurrently meet personal life demands or vice versa [11,13]. The more individuals experience job demands, such as work overload and time pressure, the more work–life conflict they experience [14,15]. While the direction of the conflict between work and life is bidirectional, the work and personal/family boundaries are easily permeable meaning that work demands tend to interfere with personal/family life to a greater extent than if the case was in reverse [11].

Research on the intersection of work with personal life has gain considerable attention in recent years, in both directions—work affecting personal life and vice versa. Work–life interference has been found to act as a “psychosocial risk factor” for ill-health and depletion of psychological health and well-being, namely, life satisfaction [16]. Some of the outcomes that have been consistently demonstrated in studies in non-academic work settings as it relates to work–life interference include nurse burnout and turnover intentions [9], absenteeism, intention to leave [17], stress, and poor work-related performance [18]. In the academic context, work–life interference has been reported to be pervasive among university faculty members, especially for female faculty due to the academic work culture that focuses on high levels of productivity and minimizes traditional/domestic roles and responsibilities [19]. Given that balance between work and personal life remains a critical issue in academia and that incompatibility bears a negative effect on important work and health outcomes, it is critical to understand its role in burnout development.

### 1.2. Burnout

Burnout is defined as a psychological syndrome that arises from continued exposure to work-related stressors, and it is characterized by emotional exhaustion, cynicism, and sensations of ineffectiveness and lack of achievement [12]. Emotional exhaustion is the prime manifestation of burnout, as it occurs when one experiences fatigue and depleted of emotional resources, resulting in cynicism or depersonalization and callousness toward others [10]. Cynicism can manifest itself as emotional resentment towards colleagues and/or the organization [20]. Common symptoms associated with burnout include chronic indecision, lack of motivation, irritability, disengagement, and withdrawal from participating in organizational operations [8].

The effects of burnout have been well documented in the organizational literature. Factors such as prolonged heavy workloads, insufficient time for personal life, or a paucity of human or material resources can increase the risks of burnout [21,22]. Among clinical nurses, burnout has been linked to decreased job satisfaction [23], turnover intentions [20], reduced work effort, and lower-quality patient care [24]. Pre-pandemic research shows, in academia, burnout has been associated with a decline in faculty members’ ability to teach [25], lack of concentration and creativity, deterioration in mental and physical health [7,26], high turnover intentions [21,25], and actual turnover [27]. A 2019 study by Alves and colleagues found that burnout had a direct negative effect on faculty members’ quality of life, regardless of their field of expertise/study. More profoundly, female academics reported to have experienced a higher likelihood of burnout and dissatisfaction because of work–life imbalance and unresolved interpersonal conflicts [28,29]. Since burnout has been closely related to clinical nurse retention and is seemly prevalent among women, it is crucial to understand the effects of burnout on nursing faculty retention factors, specifically job turnover intentions and career satisfaction.

### 1.3. Turnover Intention

Turnover intention refers to the subjective account of one’s likelihood of leaving their employment in the near future [30]. It is the last stage of cognitive withdrawal, whereby an employee takes active steps to search for alternative employment [30]. Employee turnover has a substantial impact on remaining employees and the organization in terms of the direct cost of new recruitment, selection, and training of new staff. The indirect costs of turnover include diminished workplace morale and productivity, loss of organizational knowledge, and employee demoralization [31]. According to research, an individual’s intention to leave an organization is the immediate and the most reliable and consistent predictor of actual turnover [31,32]. Organizational/institutional characteristics and collegial relationships are crucial to employee turnover. For example, in the nursing literature, factors such as the practice environment, including leadership support, collegial relationships, professional autonomy, and role conflict, were identified as key predictors of clinical nurses’ turnover intentions and eventual turnover [24,33,34].

In academia, high turnover of the faculty leads to a decline in research activities and students’ learning [35]. More profoundly, high turnover contributes to increased burnout among remaining faculty furthering the supply–demand gap in nursing faculty workforce and the general nursing clinical workforce. Studies show that female academics have the highest attrition rates due to the fact of reports of female academics often being assigned heavier teaching loads and fewer resources than their counterparts [28,29,36]. For example, female faculty often feel pressured to assume heavier student advising committee loads, especially in departments with fewer women (e.g., STEM faculty), which negatively impacts on their productivity and satisfaction [29,37]. This is particularly concerning since nursing continues to be a primarily female-dominant profession. Although a wide range of research has been conducted on faculty turnover, few studies have focused on nurse faculty. Given the current gap in nursing faculty supply and demand in Canada [38], the retention of nursing faculty should be the top priority of institutions/organizations that aim to be efficient and effective in their operations.

### 1.4. Career Satisfaction

Career satisfaction is an individual’s evaluation of an organizational/workplace factors (e.g., advancement, development, and income) relative to their own goals, expectations, and accomplishments [39]. It is an important construct in career success and commitment [40]. In academia, one key aspect of faculty career satisfaction is the nature of the work itself as it relates to teaching, research, and service obligations [41]. Another important component of satisfaction is a sense of community within the workplace and how faculty members perceive they are valued, respected, and recognized (e.g., receiving rewards, comparable salaries) by their peers and organization [42,43]. Additionally, faculty members perceived that control of their career development [41], high degrees of autonomy [44], and the challenge they take from their work [43] were significant contributing factors to their career satisfaction. Research indicates that a faculty member who had leadership support and mentor experience greater academic success and career satisfaction [45]. In turn, faculty members who mentored colleagues and students and have quality relationships had increased career satisfaction [43,46]. 

Past studies [47,48] have found that the quality of work–life of faculty have a significant impact on their satisfaction and morale. For example, a recent study correlated a lower perception of medical faculty members’ quality of life with poor physical, psychological, and social health [7]. Among faculty, the level of satisfaction in their career was a key component in their intent to leave their organization or academia. Provided that multiple factors go into faculty retention, focusing exclusively on the linkages among work–life interference, burnout, turnover intentions, and career satisfaction should better illuminate the work-and-life-related factors that lead faculty to opt-out of an institution.

### 1.5. Hypothesized Model (Specific Aims)

Based on our theoretical framework and previous research from the nursing and management literature, we predicted that higher faculty ratings of work–life interference would be associated with higher emotional exhaustion which, in turn, would be related to higher cynicism and, ultimately, increased turnover intentions and lower career satisfaction.

## 2. Materials and Methods

### 2.1. Study Design

This study adopted a non-experimental predictive design to examine the relationships described in the hypothesized study model. 

### 2.2. Participants and Settings

Nursing faculty members employed in both college and university settings in Canada were recruited to participate in this study. Inclusion criteria consisted of faculty with various appointments/positions (e.g., lecturers, assistant professor, and teaching track) in undergraduate and graduate nursing programs. Adjunct, casual, or visiting professors were excluded. Eligible participants were identified based on their institution’s online profile and were sent an email request with a link to complete a web-based structured questionnaire housed in Qualtrics. The survey consisted of several baseline characteristics along with valid and reliable instruments. The survey package included a letter explaining the study risks and benefits and strategies undertaken to ensure confidentiality and anonymity (e.g., no directly identifiable information or IP addresses). Data for this study were collected in summer 2021. Participation was voluntary and respondents could withdraw from the survey at any time prior to submitting their response. Return of a completed survey indicated consent to participate. To improve survey response rates, the Dillman [49] method was used. To maintain confidentiality, participants were randomly assigned personal identifying numbers (PIN) numbers to complete the survey anonymously (see study protocol – page 4, paragraph 2) [50]. Non-responders were sent a reminder email three weeks after the initial invitation, followed by a reminder message four weeks later to optimize response rates and to obtain an adequate sample size (≥200 participants) [51]. 

### 2.3. Instrument Validity, Reliability, and Rigor

In total, four instruments were used to measure the key variables in this analysis. All the measures were standardized questionnaires with acceptable psychometric properties and demonstrated construct validity [12,52]. The scores for each of the items were averaged to obtain an overall measure for each of the variables. 

*Interference between work and personal life* was measured using a modified version of Fisher-McAuley et al.’s [52] work interference with personal life (WIPL) scale. The WIPL is a questionnaire designed to measure directions and domains of work-personal life interference and enhancement. The 7-item scale measures the extent to which an employee’s working life has affected maintaining a work–life balance. Scale items include, “My personal life suffers because of my work” and “I often neglect my personal needs because of the demands of my work”. Responses are provided on a 7-point Likert-type scale ranging from 1 (Not at all) to 7 (Almost all the time), with lower scores indicating a better work–life balance and high scores representing work–life interference. The construct validity of the WIPL scale was established in a confirmatory factor analysis (CFA) that showed a good fit for the hypothesized factor structure (*χ*^2^ = 247, *df* = 122, CFI = 0.97, RMSEA = 0.06) [9]. Internal consistency reliability was established among nursing and business samples [9,11] with Cronbach’s α of 0.92 and 0.89, respectively. Internal scale consistency was comparable in this study (α = 0.93).

*Burnout* was measured by the emotional exhaustion and cynicism subscales of the Maslach burnout inventory-general survey (MBI-GS) [10], each consisting of 5 items. Sample items of the MBI-GS are framed as statements of job-related feelings (e.g., “I feel burned out from my work”; “I feel confident that I am effective at getting things done”) and are rated on a 6-point Likert scale from 0 = never to 6 = daily. Burnout is reflected in higher scores on exhaustion and cynicism and lower scores on efficacy, whereas the opposite pattern reflects greater engagement. Higher scores (≥3.0) on each subscale reflect burnout [12]. Previous research using the MBI-GS among nurses has demonstrated acceptable reliability and validity [9,12,53]. Cronbach’s α in the present study was 0.95.

*Intention to leave the job* was measured using a three-item scale developed by Camman et al. [54]. The items on the scale determined whether the employee is likely to voluntarily leave the organization in the near future. Respondents rated items such as, “I plan to leave this organization in the next year” on a 7-point Likert-type scale from 1 (strongly disagree) to 7 (strongly agree). This scale has demonstrated construct validity and acceptable internal consistency in samples of clinical nurses [9] and frontline nurse managers [55] with Cronbach’s α of 0.92 and 0.80, respectively. Similar Cronbach’s α are reported in this study.

*Career satisfaction* was measured using the five-item career satisfaction scale developed by Greehaus et al. [11]. Respondents indicated their level of agreement with each of the statements (sample item: “I am satisfied with the progress I have made toward meeting my overall career goals”) on a 5-point Likert scale rating from 1 (strongly disagree) to 5 (strongly agree). The mean score was computed as an index of career satisfaction, with a higher score indicating greater satisfaction. Reliability testing of the scale using a normative sample was shown to be internally consistent with a Cronbach’s α of 0.84 [56]. In the present study, the internal reliability was acceptable (α = 0.79).

### 2.4. Data Analysis

Data were downloaded from Qualtrics and analyzed using the Statistical Package for Social Sciences software (SPSS^®^) (version 25, Armonk, NY, USA) [57] and later exported to the Analysis of Moment Structures (AMOS) statistical software program (version 25) [58] for structural equation modeling (SEM) analysis. Descriptive statistics, including measures of central tendency and dispersion, were computed and the reliability of each measurement tool was tested using Cronbach’s α coefficient. Collinearity diagnostics indicated the absence of singularity or multicollinearity [51]. The hypothesized model in this study was tested using path analysis within SEM procedures in AMOS [58]. Significance levels of the direct and indirect effects in the model were estimated using Preacher and Hayes’ bootstrapping method with 5000 bootstrap samples [59] as a more robust way of testing mediation hypothesis. Statistically significant results were achieved if the 95% confidence intervals did not contain zero [60]. In SEM, a sample size of ≥200 is recommended [51] to have confidence in the goodness-of-fit tests. The index of overall fit of the hypothesized model was evaluated using the following criteria: the omnibus fit indices (e.g., chi-square, *p*-value, and chi-square/degrees of freedom ratio), the incremental fit indices (e.g., comparative fit index (CFI), Tucker–Lewis index (TLI), and the incremental fit index (IFI)) [61]. The critical value for CFI and IFI is ≥0.90 [51]. The root mean square error of approximation (RMSEA) can be considered an “absolute fit index”, with 0 indicating the “best fit” and values > 0 suggest a worse fit [51]. Values of 0.05 or below on the RMSEA are generally considered indicative of a close-fitting model. Values between up to 0.08 and 0.10 [61] are considered acceptable. However, an RMSEA ≥ 0.10 suggests a model that may have more serious problems in its specification [51]. Assessment of the above criteria are reported in Section 3.

### 2.5. Ethical Consideration

Data collection began after obtaining ethics approval from the Hamilton Integrated Research Ethics Board (#1477).

## 3. Results

### 3.1. Demographic Characteristics

Among the 1649 eligible participants invited, a total of 645 participants provided valid responses (response rate = 39.1%). Faculty mainly self-identified as female (93.6%), 83.1% were White, and 68.7% were married. The majority (81%) reported being employed in a university, and over three-quarters (76.1%) were non-tenured. Thirty-eight percent worked in a large university, and 33.3% worked in a mid-sized university. Respondents worked mostly in a full-time permanent capacity (70.2%), were either master’s (54.9%) or PhD (31.9%) prepared, and 40.9% had been at their current organization for over 10 years. An additional demographic profile of the participants is reported in Table 1.

### 3.2. Descriptive Statistics

The means, standard deviations (SDs), Cronbach’s alpha reliabilities, and the correlation matrix for the major study variables are reported in Table 2. Scores on each measure were normally distributed and all alphas were within acceptable ranges (0.79–0.95). Faculty, on average, reported moderately high levels of work interfering with life (M = 4.59, SD = 1.38) and emotional exhaustion (M = 3.68, SD = 1.68). Scores on cynicism (M = 2.91, SD = 1.44) and turnover intentions (M = 2.16, SD = 1.01) were rated slightly low. Overall, respondents to the survey were highly satisfied with their careers (M = 4.08, SD = 0.76). 

### 3.3. Test of the Hypothesized Model

**Figure 2:**
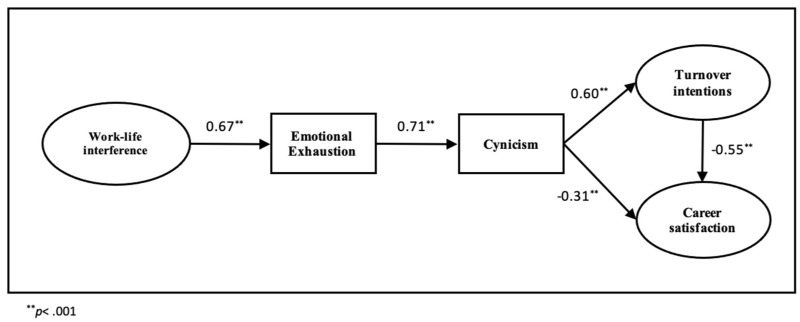
Final study model. Standardized coefficients.

The hypothesized model was supported by the model fit statistics: χ^2^_(5)_ = 7.883, *p* = 0.001, IFI = 0.99, TLI = 0.99, CFI = 0.99, and RMSEA = 0.03, indicating that the data were a good fit to the model. As hypothesized, work interference with life domains had a strong direct positive effect on emotional exhaustion (β = 0.67, *p* < 0.001) which, in turn, had a significant positive effect on cynicism (β = 0.71, *p* < 0.001). Cynicism was positively associated with turnover intentions (β = 0.60, *p* < 0.001) and negatively with career satisfaction (β = −0.31, *p* < 0.001). In addition, turnover intentions had a negative direct effect on career satisfaction (β = −0.55, *p* < 0.001). The standardized effects of coefficient in the model are illustrated in Figure 2.

## 4. Discussion

This study investigated the extent to which work demands of nursing faculty were related to their turnover intentions and career satisfaction through experiences of burnout (emotional exhaustion and cynicism). Overall, the results provide support for the hypothesized model linking faculty work–life interference with increased burnout (emotional exhaustion and cynicism) and subsequent higher turnover intentions and lower career satisfaction. We found that work–life interference had a robust positive effect on emotional exhaustion which, in turn influenced cynicism as described in the burnout theory. In addition, cynicism had both a negative effect on career satisfaction and a direct positive effect on turnover intentions, which is a phenomenon that occurs after sustained emotional exhaustion resulting from stressful working conditions [12]. Subsequently, increase in turnover intentions was shown to have led to lower career satisfaction. 

Consistent with previous research in the education and healthcare fields, work–life interference was related to high levels of emotional exhaustion [9,25,62]. The interactions between work and home (non-working) life are important for ensuring well-being, especially during COVID-19 where the lines between work and home are blurred [63]. The increasing demand for work in nursing schools and colleges has led to an increase in workload, long workhours, course overloads, additional clinical rotations, and irregular work schedules attributed to environmental distributions [64,65]. This was highlighted in a report by The National League for Nursing (NLN), indicating that nursing faculty work more than 56 hours per week with a high workload and found it challenging to achieve a work–life balance [66], especially for faculty who teach in multiple differing environments, including clinical/hospital settings that involve day, evening, and weekend hours. The findings in our study confirm this, where nursing faculty reported that working longer hours and experiencing greater work–life imbalance predisposed them to higher risk for emotional exhaustion and cynicism. 

Ongoing exposure to workplace stressors, including heavy workload and work–life interference, is the primary mechanism for developing severe burnout as confirmed by this study, where emotional exhaustion among faculty led to increased cynicism. Cynicism can be attributed to faculty members’ feelings of disrespect and anger towards their organizations or discomfort, hatred, and even shame when they think about their organizations, which may lead to decline in organizational commitment and eventual turnover [67]. In the healthcare literature, increased cynicism has been found to lead to deviant behavior, such as misappropriation, intentional wrong doings, damaging materials, and aggression, which can be damaging to the organization [68,69]. Cynicism can lead to serious disruption in the teaching–learning environment [62]. When faculty members lose trust in their organization and have increased cynicism, they are unwilling to stay with the organization, leading to a higher turnover rate [70,71]. Operating with fewer human resources results in greater dissatisfaction among existing faculty, leading to nursing faculty seeking other positions outside their current organizations and eventually leaving academia altogether [72,73]. Another study identified the factors leading to high turnover rates due to the fact of dissatisfaction including lack of extrinsic rewards, scheduling conflicts, family/work imbalance, poor collegial interaction, limited professional opportunities, praise/recognition, and control/responsibility [70,74,75]. When these factors are not being satisfied, staff become cynical and lack trust within the organization and are highly inclined to seek employment elsewhere. 

As indicated in this study, the negative implications of work–life imbalance and burnout among nursing faculty can be long lasting as it influences key workplace retention factors. While no one is immune to burnout, a US-based study found that PhD-prepared faculty experience higher emotional exhaustion compared to DNP-prepared faculty [28]. Furthermore, early career researchers are more likely to experience sustained emotional exhaustion as, historically, most nurse academics transition from a clinical background with little preparation for the complex faculty role [76], which further contributes to their vulnerability in the high-pressure academic work environment. For example, a Canadian study showed that when nurses are being recruited to work, they are not prepared for the level of mental exhaustion in relation to their perspective of the challenges of the career [77]. Specifically, lack of leadership and collegial support has been linked to increased turnover intentions [38,75], disappointment, and dissatisfaction of the career chosen and eventual turnover [38]. This extends to mid-career and senior faculty, where negative working conditions can play a key role in early retirement [28]. Increased career exits further contribute to nursing faculty shortage, which negatively impact student training, mentoring, and preparation of highly skilled nurses equipped to care for patients [65,78].

Our findings suggest that there is a need to politically address burnout, as studies in Canada and in other countries indicate that burnout can lead to emotional and physical symptoms such as an uneven chronotype [73,79,80,81], as the physical and psychological symptoms may negatively impact the mental well-being of faculty resulting in high turnover and career dissatisfaction as shown in this study. In contrast to our study findings, one study has shown that very few nursing faculty members leave their careers due to the fact of dissatisfaction, especially if they have higher education in that field [82]. While retaining such faculty is important, it is even more crucial to understand the impact of faculty dissatisfaction on their productivity, organizational commitment, morale, and workplace culture. To retain satisfied nursing faculty, academic leaders must find ways to support the development of programs and implement targeted interventions to help nursing faculty navigate work–life balance and manage stress and burnout, including setting work options for flexible work practices and maximum hours worked per week to maintain productivity [75,81,83]. Additionally, leaders should implement workplace wellness policies, commit to plans that will increase efficiency and productivity, and frequently review long-term plans to help prioritize organizational goals and objectives [84]. In creating these supportive working conditions, these leaders will enable nursing faculty to adopt empowering strategies shown to reduce burnout including prioritizing their personal health, engaging in a balanced work–life practices and personal activities, seeking peer support, and advocating for systemic change [48,85,86]. These strategies are important especially during and post-COVID-19, as burnout can have a ripple effect leading to a further shortage of registered nurses to provide high-quality care to patients and communities.

While burnout is a common phenomenon in academia, in addition to supporting previous literature [75], this study introduced and tested the mediating effects of burnout on the relationships between work–life interference, turnover intentions, and career satisfaction among nurse academics in Canada. The results afford increased understanding of nursing faculty’s experience and may provide suggestions for suitable areas for the development of interventions and policies within the organizational structure to reduce the risk of burnout and faculty leaving their positions. Given the strong evidence of the negative health and organizational effects of burnout [7] and the current nursing faculty shortage, it is particularly important to address work–life issues in effort to sustain a strong nursing workforce. 

## 5. Strengths and Limitations

This study has several strengths. For example, to our knowledge, this is among the first studies to link work–life interference, burnout, turnover intentions, and career satisfaction in the nursing or management literature in a comprehensive way among Canadian nurse academics. Furthermore, this is among the few studies reporting nursing faculty environment and related stressors during a global pandemic. The use of validated instruments to measure burnout, emotional exhaustion, and career satisfaction allows for easier comparison to other results/data reported in other national and international studies using these same measures. Additionally, the survey was anonymous which hopefully served to produce more honest, candid responses from those who completed and returned a survey. Another strength of this study is its implementation period, during summer 2021, which was in one of the peak time frames of the COVID-19 pandemic; thus, these data arguably capture the perspectives and views of nursing faculty as they practiced nursing during these unprecedented times. 

Using this cross-sectional design, we were unable to confirm causation. Therefore, the results presented should be translated as non-directional relationships [87]. Given the response rate is moderate and comparable to studies with similar samples, the findings can only be generalized to other academic institutions with similar challenges. Additionally, we did not perform comparative analysis on the academic rank and/or location for those who did not respond compared to those who did, to justify absence of selection bias. Another potential limitation is that we might have missed nursing faculty who may have a faculty appointment in a setting other than ‘nursing’; however, part of our process searched based on faculty members’ degrees (e.g., RN) rather than solely on a faculty member’s department that held their primary academic appointment.

## 6. Conclusions

This research adds to the small but crucial body of research describing the effects of work–life imbalance and burnout on faculty retention and career satisfaction. Our findings suggest that academic institutions and organizations need to pay close attention to the drivers of burnout and associated symptoms and ensure that concrete and proactive approaches and mechanisms are in place to mitigate the effects of psychological stress and burnout on faculty mental health and wellness, especially during and post-pandemic. With the current nursing practice and faculty workforce shortages, every effort must be made to create healthy work environments to retain satisfied and productive faculty, as it has subsequent effects on the quality of student training, mentoring, and quality research to advance nursing practice globally. Promoting work–life balance and workload management, including reducing teaching assignments and service commitments, providing adequate time for research activities, and pausing the tenure clock for pre-tenured faculty, is a great start to improving faculty satisfaction, retention, and career longevity. 

## Figures and Tables

**Table 1:** Frequencies for faculty demographic characteristics.

Characteristic	*n*	%
Sex		
Female	604	93.6
Male	36	5.6
Other	5	0.8
Age		
≤39 years	145	22.5
40–49 years	191	29.6
50–59 years	195	30.2
≥60 years	106	16.4
Prefer not to say	8	1.2
Highest education		
PhD	206	31.9
Masters	354	54.9
Bachelor	79	12.2
Diploma	6	1.0
Academic rank		
Lecturer	230	35.7
Assistant Professor	144	22.3
Associate Professor	82	12.7
Full Professor	88	13.6
Clinical/Sessional instructor	101	15.7
Tenure status		
Tenured	152	23.6
Tenure track	82	12.7
Teaching track	168	26.0
Non-tenure track	149	23.1
Clinical track	92	14.3
Employment status		
Full-time permanent	453	70.2
Full-time temporary	75	11.6
Part-time	117	18.2
Years worked in current organization		
≤1 year	45	7.0
2–5 years	200	31.0
6–10 years	136	21.1
>10 years	264	40.9
Hours worked per week		
≤35 h	85	13.2
36–40 h	122	19.0
41–45 h	121	18.8
>46 h	314	48.9

**Table 2:** Correlations, means, standard deviations, and reliabilities of the major study variables.

Study Variable	M	SD	Range	α	1	2	3	4	5
1. Work–life interference	4.59	1.38	1–7	0.93	1	0.65 **	0.47 **	0.28 **	−0.33 **
2. Emotional exhaustion	3.68	1.68	0–6	0.94		1	0.71 **	0.39 **	−0.41 **
3. Cynicism	2.50	1.89	0–6	0.93			1	0.51 **	−0.55 **
4. Turnover intentions	2.17	1.02	1–5	0.76				1	−0.49 **
5. Career satisfaction	4.08	0.761	1–5	0.79					1

M = mean; SD = standard deviation; α = Cronbach’s alpha. ** Significant = *p* ≤ 0.001.

## Data Availability

Research data are not publicly available due to the restrictions (e.g., contains information that could compromise the privacy of research participants) and in accordance with the ethics agreement.
